# Supplementary material for: Improving the Aerobic Capacity in Fingerlings of European Sea Bass (Dicentrarchus labrax) through Moderate and Sustained Exercise: A Metabolic Approach
Source: Animals (Basel). 2024 Jan 16;14(2):274. doi: 10.3390/ani14020274 (PMC10812480; doi:10.3390/ani14020274)

Improving the Aerobic Capacity in Fingerlings of European Sea Bass (*Dicentrarchus labrax*) through Moderate and Sustained Exercise: A Metabolic Approach

Miquel Perelló-Amorós , Jaume Fernández-Borràs , Shengnan Yu, Albert Sánchez-Moya, Daniel García de la serrana, Joaquín Gutiérrez and Josefina Blasco *

Supplementary materials

Figure S1: Reproducibility of 2D-gels: Western blots images and their respective total protein

The supplementary Figure S1 recopilates the original Western blots images and their respective total protein (Revert) stains used in the present work. To fit all the samples, each antibody was incubated over two membranes (named 1 and 2) and the specific immunoreactive bands that were quantified are highlighted by green arrows. The molecular weight marker bands used to determine the inmunoreactive bands are highlighted in red. The distribution of the samples within each gel is the same for all of them (see the well numeration in the CS WM images). For each membrane, from left to right: wells 1 and 15 correspond to molecular weight marker, well 2 correspond to a random simples choosen as a loading control to assess variability among membranes, wells 3 to 8 correspond to control fish and wells 9 to 14 correspond to exercised fish. For the preparation of the figures, two representative bands from the immunoreactive blots and the corresponding total protein stains were cropped and identified between brackets in the title of each blot. The cropped bands from the total protein stain used for the figures are highlighted by green brackets.

CS WM (Membrane 1 - CT: wells 3-4, EX: wells 9-10)

Membrane 1

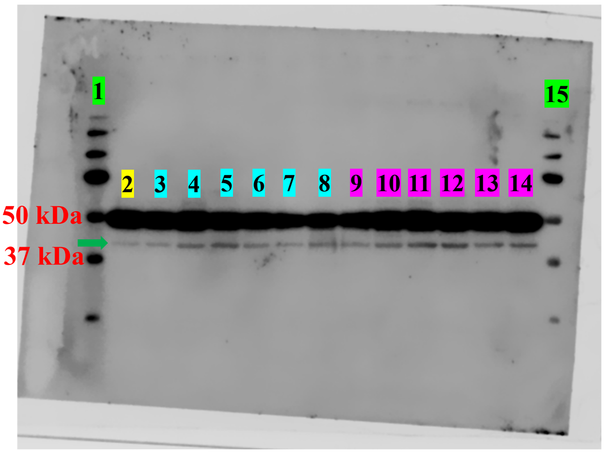

Membrane 2

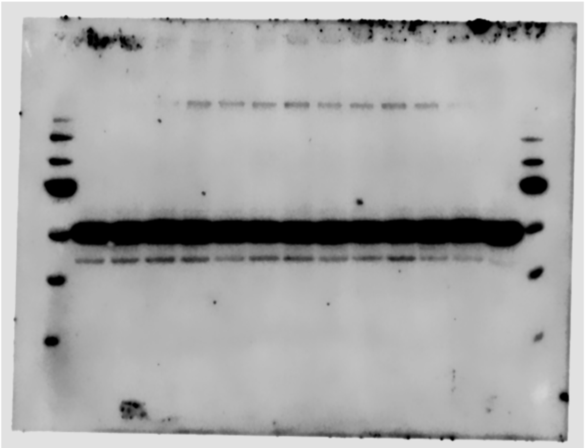

Revert CS WM

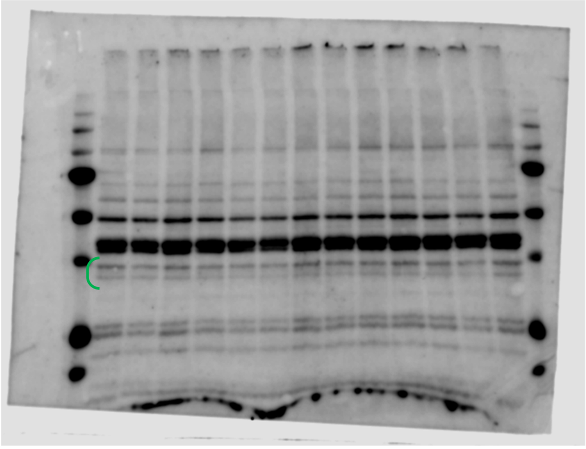

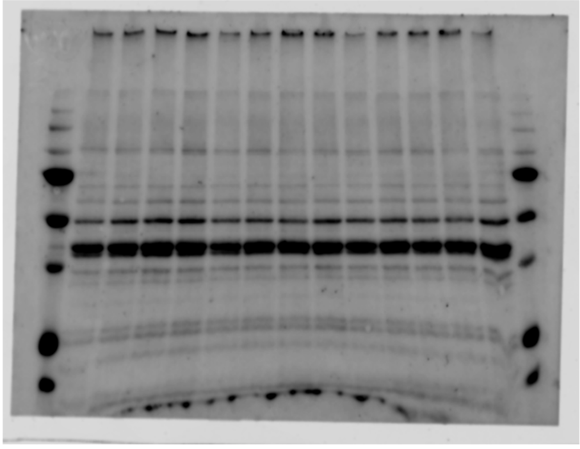

CS RM (Membrane 1 - CT: wells 6-7, EX: wells 10-11)

Membrane 1

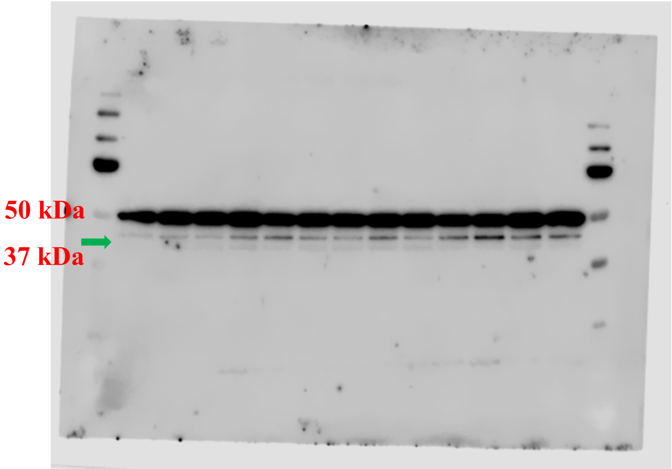

Membrane 2

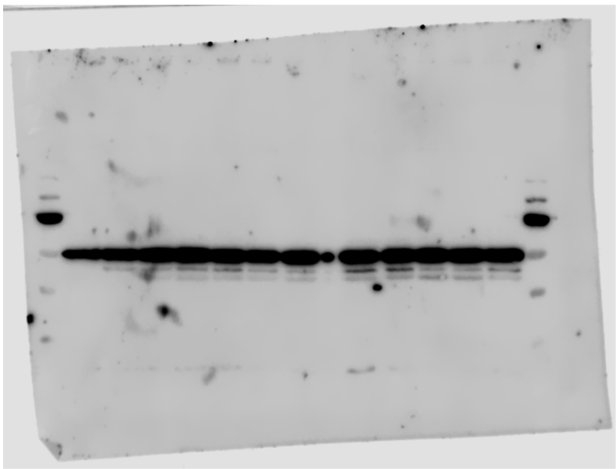

Revert CS RM

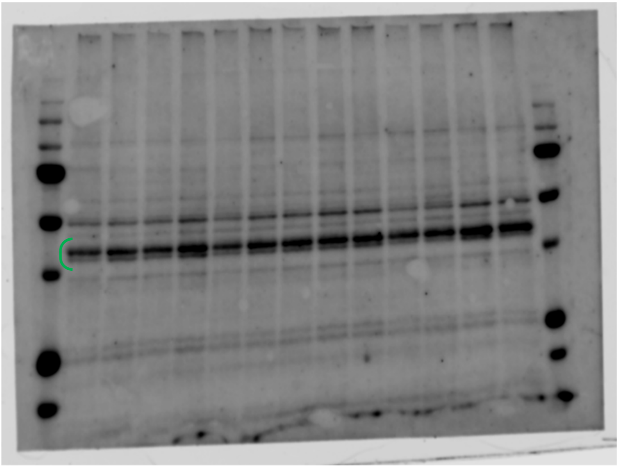

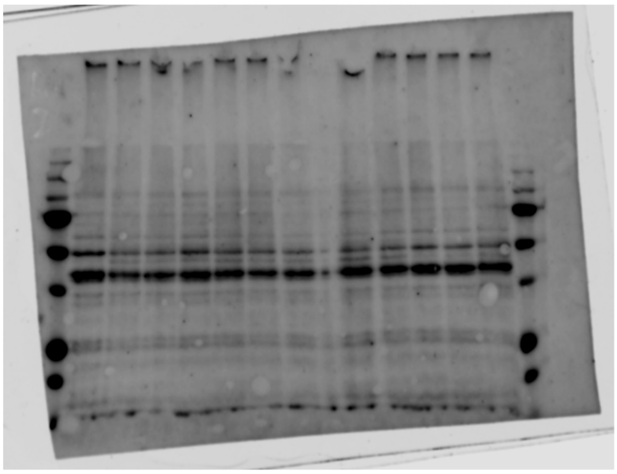

COX WM (Membrane 1 - CT: wells 5-6, EX: wells 8-9)

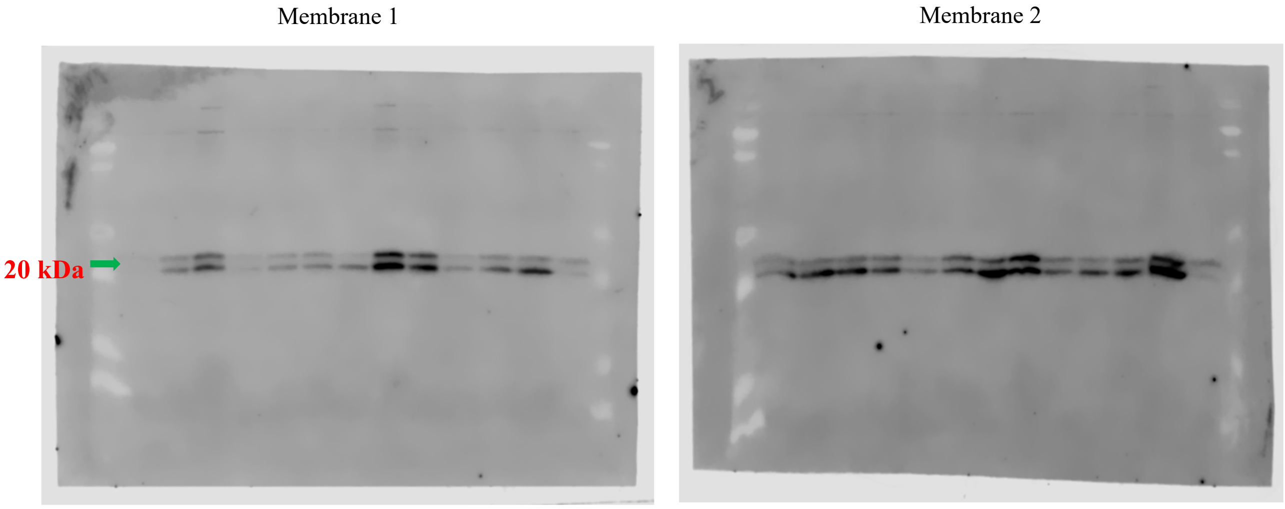

Revert COX WM

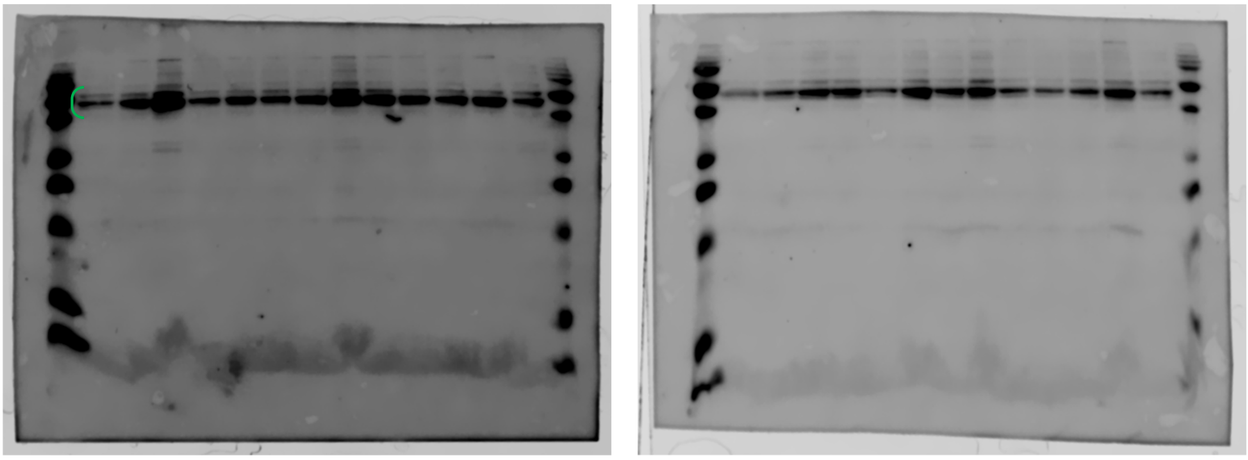

COX RM (Membrane 1 - CT: wells 4-5, EX: wells 8-9)

Membrane 1

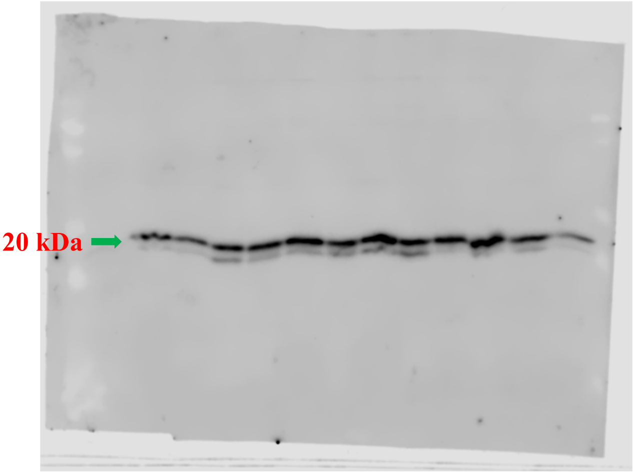

Membrane 2

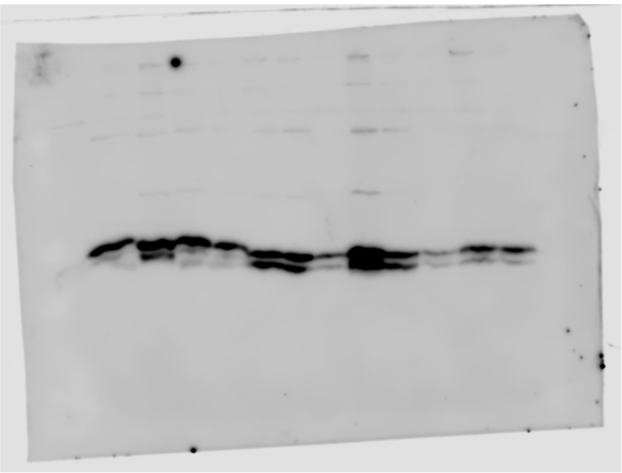

REVERT COX RM

Membrane 1

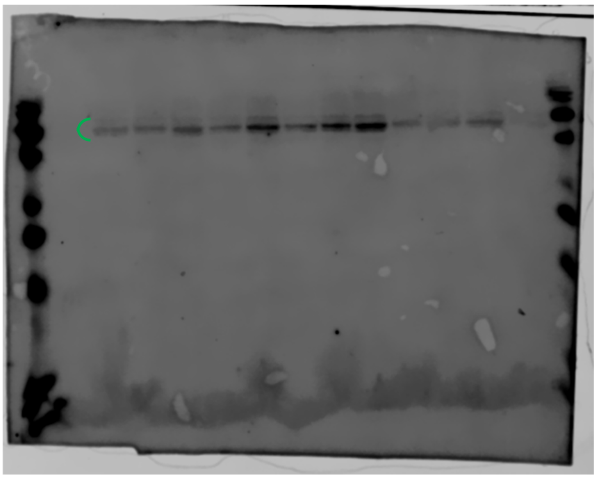

Membrane 2

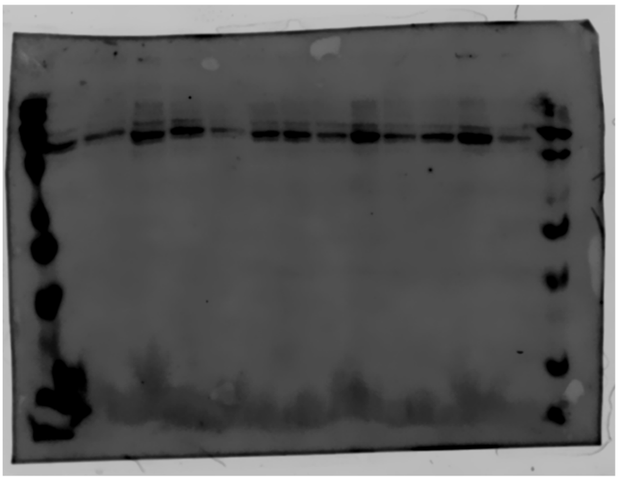

UCP3 WM (Membrane 1 - CT: wells 5-6, EX: wells 10-11)

Membrane 1

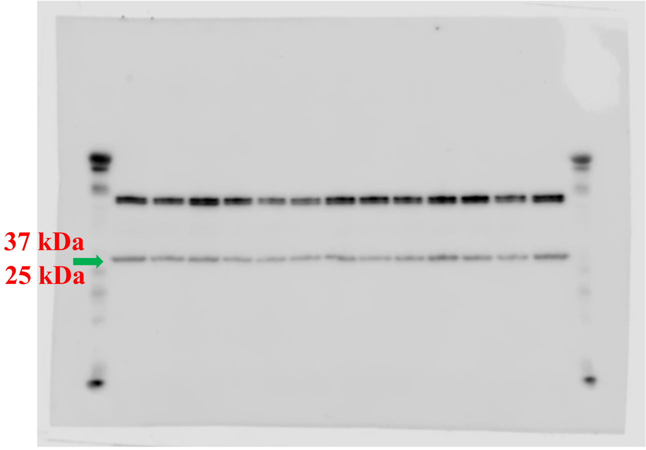

Membrane 2

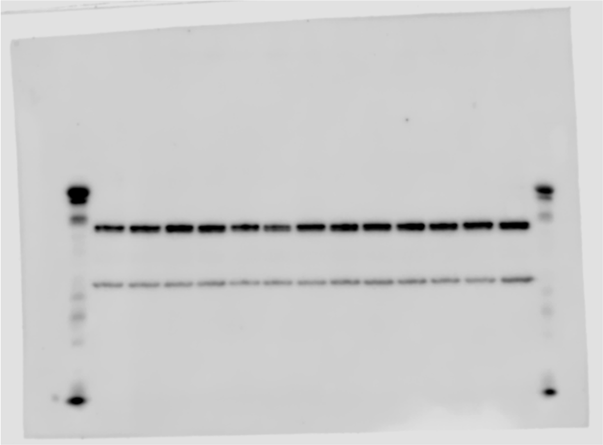

REVERT UCP3 WM

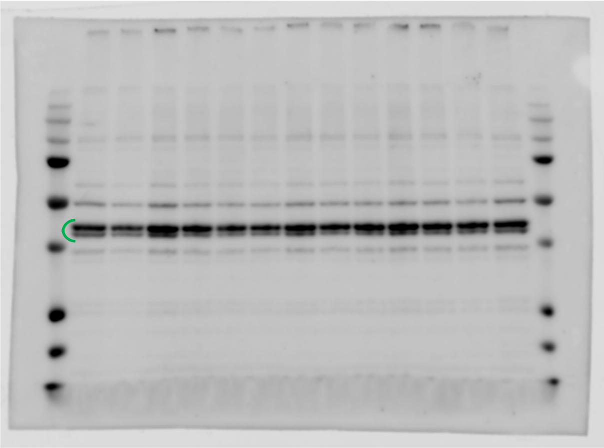

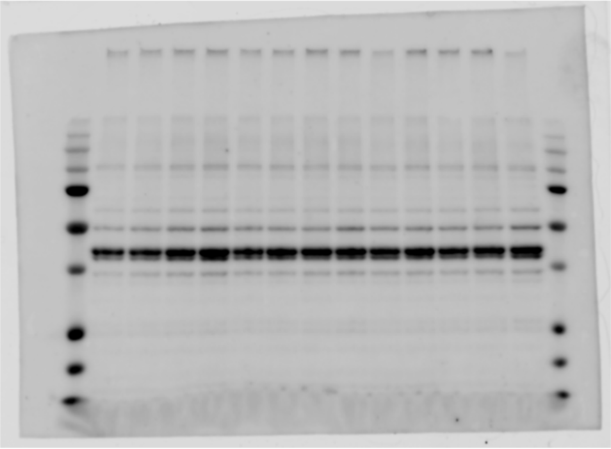

UCP3 RM (Membrane 1 - CT: wells 3-4, EX: wells 8-9)

Membrane 1

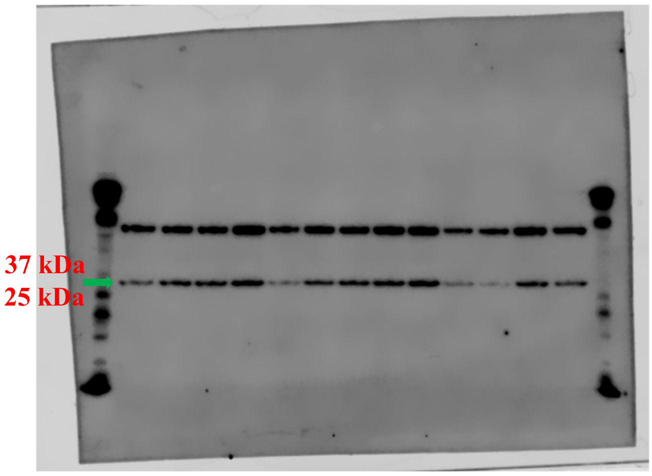

Membrane 2

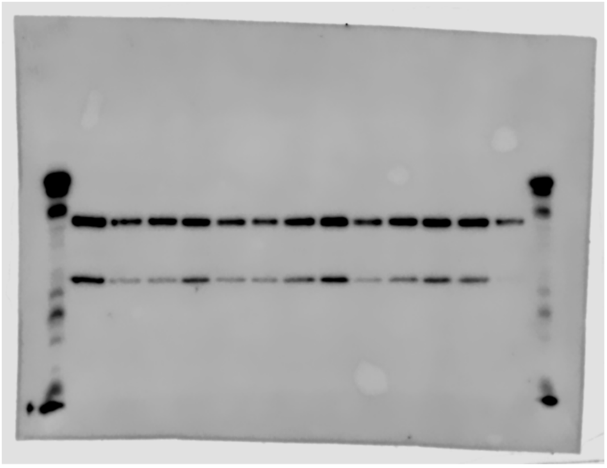

REVERT UCP3 RM

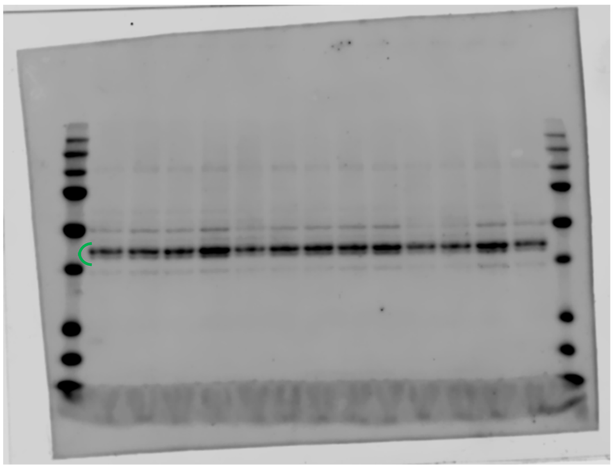

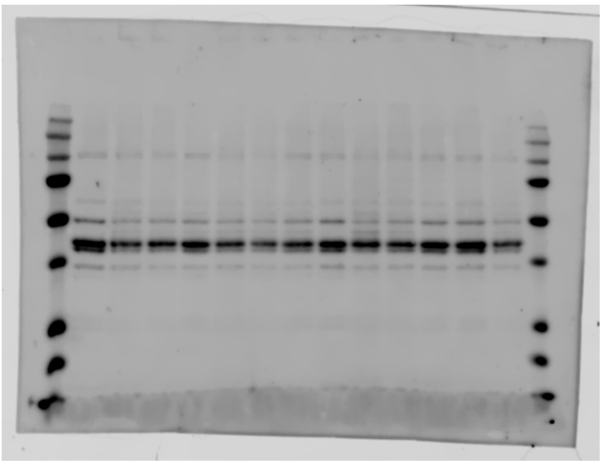

Supplement: Supplementary file 1 [file animals-14-00274-s001.zip › animals-2799937-supplementary.pdf]
